# Identification of housekeeping gene for future studies exploring effects of cryopreservation on gene expression in shrimp

**DOI:** 10.1038/s41598-025-95258-6

**Published:** 2025-04-01

**Authors:** Yen-Po Chen, Chiung-Chih Hu, Sujune Tsai, Zhi-Hong Wen, Chiahsin Lin

**Affiliations:** 1 Department of Obstetrics and Gynecology, Kaohsiung Armed Forces General Hospital, Kaohsiung, 80284 Taiwan; 2 Department of Obstetrics and Gynecology, Tri-Service General Hospital, National Defense Medical Center, Taipei, 90055 Taiwan; 3 Graduate Institute of Marine Biology, National Dong Hwa University, Pingtung, 974301 Taiwan; 4 Department of Post Modern Agriculture, Mingdao University, Chang Hua, 52345 Taiwan; 5 Department of Marine Biotechnology and Resources, National Sun Yat-Sen University, Kaohsiung, 804 Taiwan; 6 National Museum of Marine Biology and Aquarium, 2 Houwan Rd., Checheng, 94450 Pingtung Taiwan

**Keywords:** Housekeeping gene, Cryopreservation, Shrimp, Chilling, Gene expression, Biotechnology, Cell biology, Developmental biology, Molecular biology, Physiology

## Abstract

Few studies have investigated the subcellular effects of low temperature on gene expression in shrimp and most other crustaceans. Before gene expression analysis is conducted, suitable housekeeping genes (HKGs) must be confirmed to account for differences in reverse transcription process efficiency among samples. Thus, this study aimed to verify five frequently used HKGs, namely 18S ribosomal RNA (18S rRNA), ATPase, histone 3, β-actin, and glyceraldehyde 3-phosphate dehydrogenase (gapdh) for use in experiments for assessing the molecular-scale effects of cryopreservation on coral banded shrimp (*Stenopus hispidus*) embryos. To conduct chilling studies, we subjected *S. hispidus* embryos to incubation at either 26 °C (control) or 5 °C for 0, 4, 8, 16, or 32 h. The software tools GeNorm, NormFinder, and Bestkeeper were employed to identify the most suitable HKG. GeNorm identified histone 3 and 18S rRNA as the most stable genes. By contrast, NormFinder determined that 18S rRNA is a stable gene for eye-formation and pre-hatch stage samples. Finally, Bestkeeper determined that gapdh and β-actin are the most suitable genes. This study is the first to identify suitable HKGs for studying shrimp embryos at low temperatures. Its findings can aid future research on evaluating the effects of cryopreservation on gene expression in crustaceans.

## Introduction

Genes play a crucial role in shaping the hereditary characteristics of animals. The process of regulating gene expression in cells causes them to develop distinct shapes and functions^1^. The genes referred to as housekeeping genes (HKGs) are unaffected by experimental settings, endogenous variables, or external factors^2,3^. In molecular biology research, HKGs are commonly used as internal controls in gene expression studies using real-time reverse transcription polymerase chain reaction (RT-PCR). These genes are present and stable in the cells and tissues of various organisms, including crustaceans, fish, mammals, oysters, and Antarctic ice algae. Numerous studies have evaluated HKGs^1,3–13^.

The study of gene expression is becoming increasingly crucial in research on cell damage, disease causes, and therapies^1,14,15^. Gene expression may be utilized to assess the function of cells or tissues inside an organism^16^. For cryopreservation research, regardless of the method used (e.g., controlled slow cooling^17^, multi-step freezing^18,19^, and ultra-fast vitrification^20–24^, the occurrence of chilling injury or ice crystallization in cells poses a potential risk^22,25–27^. When ice crystallization occurs, cells may become ruptured or damaged in other forms^28,29^, leading to compromised survival after thawing^20,30^. Furthermore, other than being used to assess the survivability of organisms and the physical characteristics of their cells through the use of electron microscopes, which are commonly employed to examine cellular conditions^31,32^, DNA damage and changes in gene expression can also serve as indications for cell apoptosis^33,34^. Multiple studies have suggested that the preservation and survival of cells is determined by the integrity of their DNA and the genetic alterations that occur, both before and after cryopreservation^35–39^. Nevertheless, few studies have investigated the effect of cryopreservation on gene expression. Researchers have attempted to detect the DNA damage resulting from cryopreservation in various aquatic organisms, such as coral^40,41^, jellyfish^42^, shrimp^43–45^ and fish^46–49^.

RNA is synthesized through transcription from DNA, resulting in the formation of a single-stranded helical structure. The length and content of RNA sequences vary, and they can reflect the expression of individual genes. The extraction of total RNA from samples is often performed to assess gene expression^50^. Quantitative real-time polymerase chain reaction (PCR) has been used with fluorescence technology to evaluate the quantity of a product in terms of the correlation between the concentration of a sample and its corresponding Ct value. Because of its high sensitivity, this technology has been increasingly applied for quantitative gene expression in recent years^4,11,51^. To accurately detect gene expression in samples through quantitative real-time PCR, internal HKGs have been employed to normalize target genes^4,8,52^.

Gene transcription has a considerable effect on cell development. HKGs, such as β-actin, glyceraldehyde 3-phosphate dehydrogenase (gapdh), and ef1-α, are genes that perform specialized tasks in cells. β-actin is associated with cytoskeleton function, gapdh is involved in glycolysis, and ef1-α aids in the translation of RNA into protein^1,3,14^. In general, HKGs should exhibit a consistent level of expression, even when they are subjected to various treatment conditions. Nevertheless, a growing body of evidence suggests that HKGs exhibit different levels of expression under different conditions^53^. The incorrect use of HKGs may lead to notable performance problems and incorrect data interpretation. Therefore, the use of stable HKGs is crucial in gene expression research.

Several analytic algorithms, such as GeNorm, NormFinder, and Bestkeeper^2,8,10,54^, can be employed to identify suitable HKGs. GeNorm computes the average pairwise variance between a specific gene and all other control genes^55,56^. Normfinder evaluates the variability within and between groups for each gene relative to other genes, and the gene exhibiting the lowest level of fluctuation is regarded as the most stable^57,58^. Bestkeeper assesses the variability of raw Ct values among multiple genes, and genes with standard deviations of > 1 are considered to be inconsistent^10,59^. Lin et al. 2009^1^ and Chong et al. 2017^60^ identified the optimal HKGs for studying the effects of low temperature on zebrafish embryo cells and coral dinoflagellate symbiont Symbiodiniaceae. These genes were analyzed using the three aforementioned algorithms. Similarly, Leelatanawit et al. 2012^8^ and Dhar et al. 2009^4^ used the same analytical approaches to identify the most suitable HKGs for studying reproductive and immune-related gene expression in shrimps.

*S. hispidus* is a much sought after marine shrimp species that is often found in the Indo-Pacific area, the Red Sea, and the Western Atlantic Ocean^61,62^. This organism has a white physique adorned with red stripes, prominent pincers, and elongated, thin white sensory appendages^63^. The larvae of *S. hispidus* undergo serval developmental phases until they settle in the benthos. *S. hispidus* can eliminate external parasites from their hosts, such as corals and anemones^64^, and they feed on the damaged tissue and food particles found on reef fish^65–67^. These organisms are visually appealing and perform the beneficial tasks of cleaning and scavenging within aquarium tanks^68^. In addition, they act as a biocontrol agent in aquaculture, effectively reducing the number of parasites in fish tanks^69^. *S. hispidus* is highly sought after in the marine aquarium trade and ornament industry because of its vibrant hue. However, they face substantial risks from human activities (e.g., fishing) and natural calamities.

Therefore, this present study aimed to identify the optimal HKGs for conducting low temperature research on the embryos of the banded coral shrimp *S. hispidus* through the use of quantitative real-time PCR.

## Material and methods

### Collection of S. hispidus

**Fig. 1:**
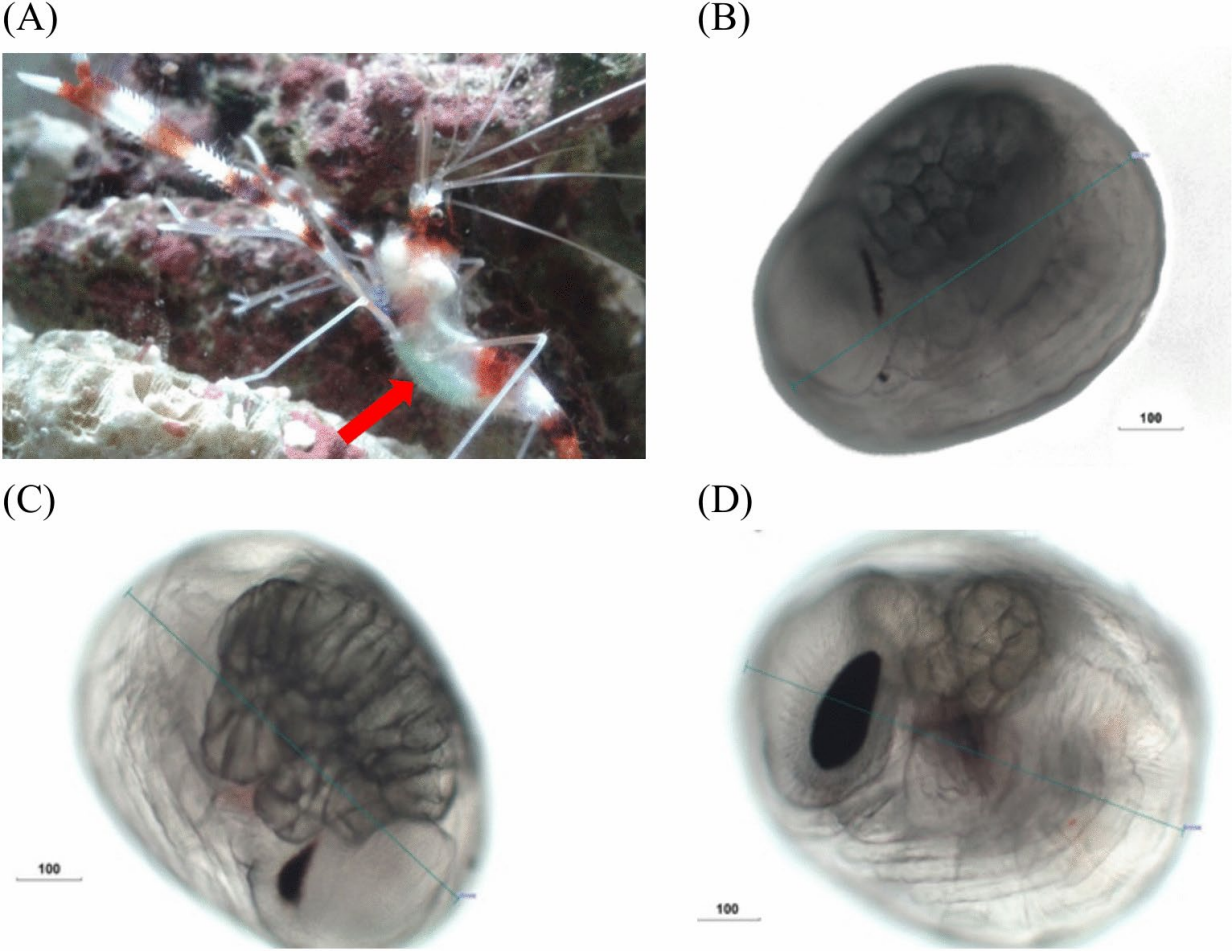
Early stage of *S. hispidus* embryos. The color of the embryo is blue–green, as indicated by the arrow. (**A**) The embryogenesis of *S. hispidus*. (**B**) Eye spots emerge throughout eye formation. (**C**) A slow heartbeat can be observed during the heartbeat stage. (**D**) Prior to hatching, the embryo’s eye spots enlarge, its green color intensifies, its heart rate quickens, and its organs become fully developed. Unit: μm.

*S. hispidus* was acquired from local aquariums in Pingtung, Taiwan. After the males and females were matched, they were placed into the tanks equipped with a natural seawater filtration system. The temperature was consistently maintained at 26 °C ± 2 °C, and the salinity was constant at 35‰ ± 1‰. The tanks were also subjected to a photoperiod with 10 h of light and 14 h of darkness. The Antarctic krill was provided twice daily at a fixed time. The male and female *S. hispidus* were paired for reproduction in the tank. After 2 weeks, the female *S. hispidus* carried blue–green embryos among its moving appendages throughout the initial stages of fertilization (Fig. 1). Under optimal temperature conditions, embryos of *S. hispidus* were produced, on average, within 25 days. During the early stage, the embryo is initially blue–green in color (Fig. 1). Subsequently, its color gradually changes to have a greener hue. Embryonic development can be divided into three distinct phases, namely the phase of eye formation, which is characterized by the appearance of eyespots (Fig. 1B); the phase of heartbeat initiation, which is marked by the onset of a weak heartbeat (Fig. 1C); and the pre-hatching phase, during which organs become fully developed (Fig. 1D). A light microscope (Olympus CX31, Japan) was used to study each stage of embryonic development.

### Chilling

A pool of 100 embryos (sourced from at least 3 individual shrimps) from each developmental stage and tested condition was placed in 1.5-mL Eppendorf tubes and incubated with filtered seawater. The embryos were then immediately transferred to a temperature-regulated dry bath (CLUBIO cb-1502, Medclub, Taiwan) set to 5 °C for durations of 4, 8, 16, and 32 h. A control group was maintained at room temperature (26 °C) for comparison. Three replicates were performed for each group, resulting in a total of 15,000 embryos (sourced from at least 9 individual shrimps) used in this study. To date, successful cryopreservation of shrimp embryos has not been achieved. A primary limiting factor is their pronounced sensitivity to chilling, particularly the sharp increase in sensitivity at subzero temperatures^70^. For this reason, 5 °C was selected as the chilling condition. At 5 °C, the embryos exhibited higher chilling sensitivity compared to temperatures above 5 °C, yet this temperature did not induce significant mortality when compared to lower temperatures, such as 0 °C^70^. The samples were then labeled and stored at a temperature of − 80 °C in a freezer for subsequent RNA extraction.

### Primer design and sequencing

The genes examined in this experiment were 18S ribosomal RNA (18S rRNA), sodium–potassium ATPase (NaK), histone (H3), beta-actin, and gapdh (Table 1). The National Center for Biotechnology Information (NCBI) website was used to obtain the sequenced mRNA sequence of *S. hispidus*. This sequence was used as a reference for designing the 18S rRNA (AY743957), ATPase (JF346359), and histone 3 (FJ943457) genes. The beta-actin primer was designed for quantitative real-time PCR analysis involving the use of comparable sequences from *Penaeus monodon*, *Litopenaeus vannamei*, and *Fenneropenaeus chinensis*. The primers for gapdh were designed on the basis of the genomic sequences of *Penaeus monodon*, *Marsupenaeus japonicus*, and *Rimicaris exoculata*. Nevertheless, the gene sequences of beta-actin and gapdh, which were highly similar, were compared using various sequences. Subsequently, the primers were designed using Vector NTI (Version.10; Thermo Fisher Scientific, USA) software. The products were verified through PCR by using the Gene-SpinTM 1–4-4 DNA Purification Kit-V2 Kit (Protech Technology, Taipei, Taiwan). The gel was cut to obtain the purified products and subjected to sequencing. Subsequently, the Roche Universal Probe Library Assay Design Center was referenced to design primers that met the experimental criteria for investigating the five gene sequences. The primers used in this investigation were purchased and synthesized by GeneMark (Taiwan) Company (Table 1). The completed primers with the KAPA2GTM Fast HotStart ReadyMix (2X) (Cape Town, South Africa) reagent were used to perform PCR and confirm the product before proceeding with subsequent experiments.

**Table 1:** Primer sequences used in this study.

Genes name	Abbreviation	Function	Primer sequence	Product length (bp)	Accession number
18S ribosomal RNA	18S rRNA	Making protein	F5′-CGGCTTGATCCGAACACTAC-3′ R5′-CAAACAAGGCCTGCTCTGA-3	120	AY743957
Sodium–potassium	ATPase (NAK)	Active transport	F5′-CGCTTTGTTGGTCTCATGTC-3′ R5′-CAGACCTGCACTTAGCAACG-3′	76	JF346359
Histone	H3	DNA folding	F5’-CTCCGTTTCCAGTCTTCTGC-3’ R5′-CCGACAAGGTAGGCTTCTGA-3	62	FJ943457
Beta-actin	β-actin	Cell motility and structure	F5′-TGTACCCTGGTATTGCTGACC-3′ R5′-TCGGGAGGAGCAATGATCT-3′	88	KT218526
Glyceraldehyde 3-phosphate dehydrogenase	gapdh	Glycolysis	F5′-AGAAGGCCTCTGCCCATT-3′ R5′-GCAGAGGGTGCAGAAATGAC-3′	61	KT072628

PCR was conducted under the conditions as follows: initial denaturation at 95 °C for 5 min, followed by denaturation at 95 °C for 30 s, annealing at 60 °C for 30 s, and extension at 72 °C for 10 s. In total, 40 cycles were implemented under these three settings, with the final temperature set at 4 °C. The duration of each cycle was 5 min, and the temperature was then adjusted to 16 °C to ensure the preservation of cDNA.

### RNA extraction and cDNA synthesis

We extracted total RNA using TRIzol (Roche, Germany) by applying a modified version of the manufacturer’s protocol. Initially, chilled embryos were equilibrated with 250 μL of TRIzol reagent (Omics Bio, Taiwan; Roche, Germany) at room temperature for 10 min. After the embryos were cut and fragmented, they were mixed with 50 μL of chloroform (Sigma, USA) at room temperature for 10 min. The mixture was then centrifuged at 4 °C for 15 min at 12 000 × *g*. The resulting supernatant was carefully transferred to a sterile Eppendorf tube. The TRIzol and chloroform treatments were repeated. Next, 500 μL of isopropanol (MERCK, Germany) was added and mixed thoroughly with a pipette for 1 min. The mixture was centrifuged at 4 °C for 15 min at 12 000 × *g* after 10 min. The supernatant was removed after centrifugation, and 1 mL of 75% ethanol (Nihon Shiyaku, Japan) was added to generate a white precipitate. Centrifuging at 4 °C for 5 min (12 000 g) was performed to disperse the precipitate. To remove phenol, washing was performed three times. The resulting ethanol residue was removed with a centrifugal evaporator (CentriVap; Labconco, USA). Finally, 16–20 μL of DEPC-treated distilled water was added to dissolve the RNA pellet. An SSP-3000 nanodrop spectrophotometer (Infinigen Biotech, USA) was used to assess RNA quality and concentration. UniRegion Biotech gel electrophoresis on 1% agarose in tris–acetate-EDTA (TAE) buffer was performed to assess and verify RNA integrity. RNA quality was validated by measuring the absorbance ratio at 260/280, which was determined to be 1.8–2.0. For post-quantification, 1 μg of total RNA was added to each reverse transcription process by using Takara Biosystems’ TaKaRa Primescript Master Mix. To obtain a 10 μL volume, the RNA template was mixed with 2 μL of MasterMix and PCR-grade water in the correct proportion. The PCR protocol is as follows. After the temperature was maintained at 37 °C for 30 min, it was increased to 85 °C for 5 s and subsequently maintained at 4 °C. Quantitative real-time PCR studies were performed using cDNA stored at − 20 °C.

### Establishing standards for quantitative real-time RT-PCR

HKG standards were established using standard PCR with Hotstart Mix (Kapa Biosystems, USA) per the manufacturer’s instructions. In summary, a 12.5-μL Hotstart Mix, 2-μL cDNA template, and 2.5 μL of each primer were added into an Eppendorf tube. The reaction mixture was diluted with PCR-grade water to 25 μL. The PCR process comprised 5 min of denaturation at 95 °C, 60 s of annealing, 10 s of extension, and 5 min of final extension. The PCR result was separated through gel electrophoresis on a 1.5% agarose gel in TAE solution. Blue-light epi-illuminators (SMOBIO, Taiwan) were employed to carefully slice targeted cDNA bands. An AxyPrep DNA Gel Extraction Kit (USA) was used to extract cDNA per the manufacturer’s instructions. Gel pulp was centrifuged in a 1.5-mL Eppendorf tube. Gel solubilization buffer was introduced as gel pulp into the tube three times. The gel was fully dissolved by vortexing the samples in a 75 °C water bath. It was dissolved in 100-μL propanol and combined with gel solubilization buffer. The samples were mixed thoroughly and placed in a new Eppendorf tube with filters. For 1 min, the tubes were centrifuged at 12 000 × *g*. After the filtrate was discarded, 500 μL of wash buffer was added to the tube. The samples were centrifuged for 30 s under the same conditions. Subsequently, 700 μL of desalting buffer solution was added to the tube after discarding the liquid from the filter. The samples were centrifuged for 30 s, after which the centrifugation duration was extended by 1 min. Finally, centrifugation was performed until the buffer was removed. After moving the filter column to a new Eppendorf tube, 20 μL of DEPC-treated water was used to extract cDNA from the membrane’s center; this procedure was performed for 1 min. The material was centrifuged for 1 min at 12 000 × *g*. The nanodrop spectrophotometer was used to measure the concentration of the purified and recovered PCR products, which were diluted to 2 ng/μL.

### Quantitative real-time PCR

All samples were subjected to conventional PCR testing before real-time PCR to verify that the primers were suitable for the shrimp samples. In accordance with the instructions provided by Kapabiosystems (USA), all sample groups, including the control group, were subjected to PCR assays. To obtain a final volume of 25 µL, PCR-grade water was mixed with 12.5 µL of ready mix, 2 µL of a specific primer, and 2 µL of template cDNA. The PCR mixture was then added to the thermal cycler per the protocol for establishing real-time PCR standards. A 1.5% agarose gel was loaded with the completed PCR result in TAE buffer for gel electrophoresis. Subsequently, ultraviolet light was used to examine the gel. As revealed in Table 3.1, the primers were detected in the band size that matched the primer.

Quantitative real-time PCR was performed on 100-fold diluted cDNA samples. Specifically, 0.5 mL of a 20-µM specific primer and 10 µL of SYBR FAST qPCR Master Mix (KAPA Biosystems, USA) were added to the diluted cDNA. The combination had a total volume of 20 µL. This method was employed to determine whether the potential HKGs’ expression was consistent across all samples. Rotor-Gene quantitative real-time PCR system (QIAGEN, Germany) was used to perform the experiment. The cDNA was diluted in a tenfold series to establish standards for each experiment. This step was performed to determine the gene concentration and optimize the threshold cycle. The experimental parameters applied for the reaction were as follows: one 5-min cycle at 95 °C, forty 10-s cycles at 98 °C, and one 20-s cycle at 60 °C. Melting curves were obtained at the end of each experiment to verify primer accuracy and identify unwanted PCR products.

### Statistical analysis

For this experiment, three biological samples were generated, and tests for each set of biological samples were conducted in triplicate. The average relative expression of each sample was calculated. The data were subjected to analysis of variance involving Games–Howell post hoc tests. Whenever necessary, the analyzed data were logarithmically transformed. This analysis was performed using SPSS and Microsoft Excel. Additionally, the GeNorm algorithm was used to identify the most stable pair of genes among the analyzed genes. Furthermore, the NormFinder algorithm was employed to assess the stability of each gene individually. The Bestkeeper algorithm was used to examine the variation in raw Ct values among the analyzed genes. Values with standard deviations greater than 1 were regarded as inconsistent. Each HKG was examined to determine the stability of its expression in fresh embryos at various stages and in fresh and chilled embryos at various stages. The stability of gene expressions at various phases of development was also examined using GeNorm, NormFinder, and Bestkeeper.

## Results

### Primer specificity

**Fig. 2:**
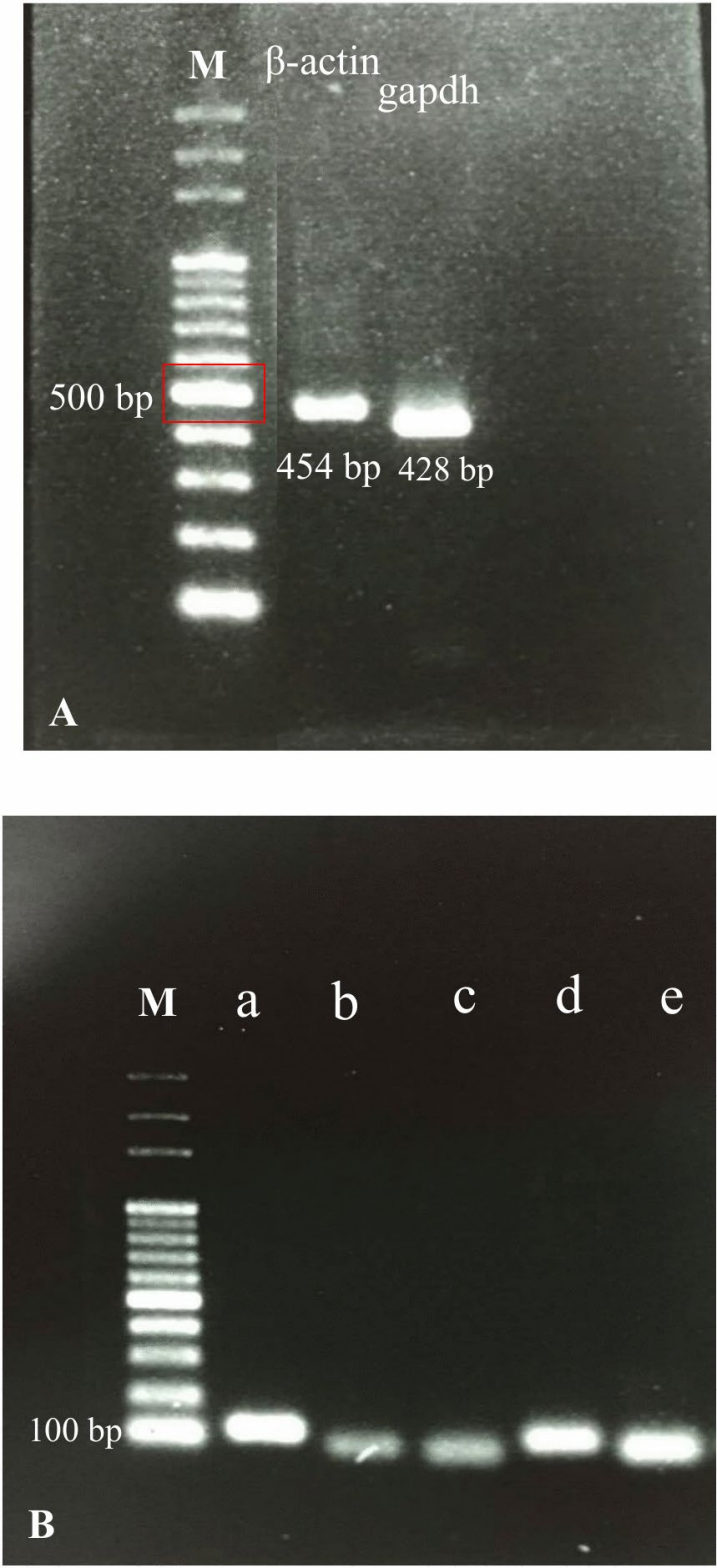
(**A**): Specificity assessment of primers β-actin (454 bp) and gapdh (428 bp) prepared for conducting multiple sequence analysis. (**B**) Specificity testing of primers for five genes assessed in the present study: a. 18S ribosomal RNA (120 base pairs); b. ATPase (76 base pairs); c. histone 3 (62 base pairs); d. β-actin (88 base pairs); e. gapdh (61 base pairs). M: marker.

The present study assessed a collective of five genes, namely 18S rRNA, ATPase, histone 3, β-actin, and gapdh. Because the β-actin and gapdh genes in *S. hispidus* were not sequenced, they had to be redesigned using sequences from other shrimp species. Figure 2A displays the specificity results of the two primers that were designed after they were compared with each other. The β-actin gene’s amplicon size was 454 base pairs, whereas the gapdh amplicon size was 428 base pairs. When the specificity of the primers was verified, the gel products were dissected for purification. Figure 2B displays the selectivity of the five gene primers examined in the present study for *S. hispidus*. The markings in the image, labeled a, b, c, d, and e, represent 18S rRNA (120 base pairs), ATPase (76 base pairs), histone 3 (62 base pairs), β-actin (88 base pairs), and gapdh (61 base pairs), respectively. The gel electrophoresis bands were indicative of the sizes of their respective primers. Figure 2A,B reveal the specificity of these five primers for *S. hispidus*. These primers were subsequently employed in quantitative real-time PCR analysis to reduce experimental error.

### Quantitative real-time PCR efficiency and Ct value analysis

**Fig. 3:**
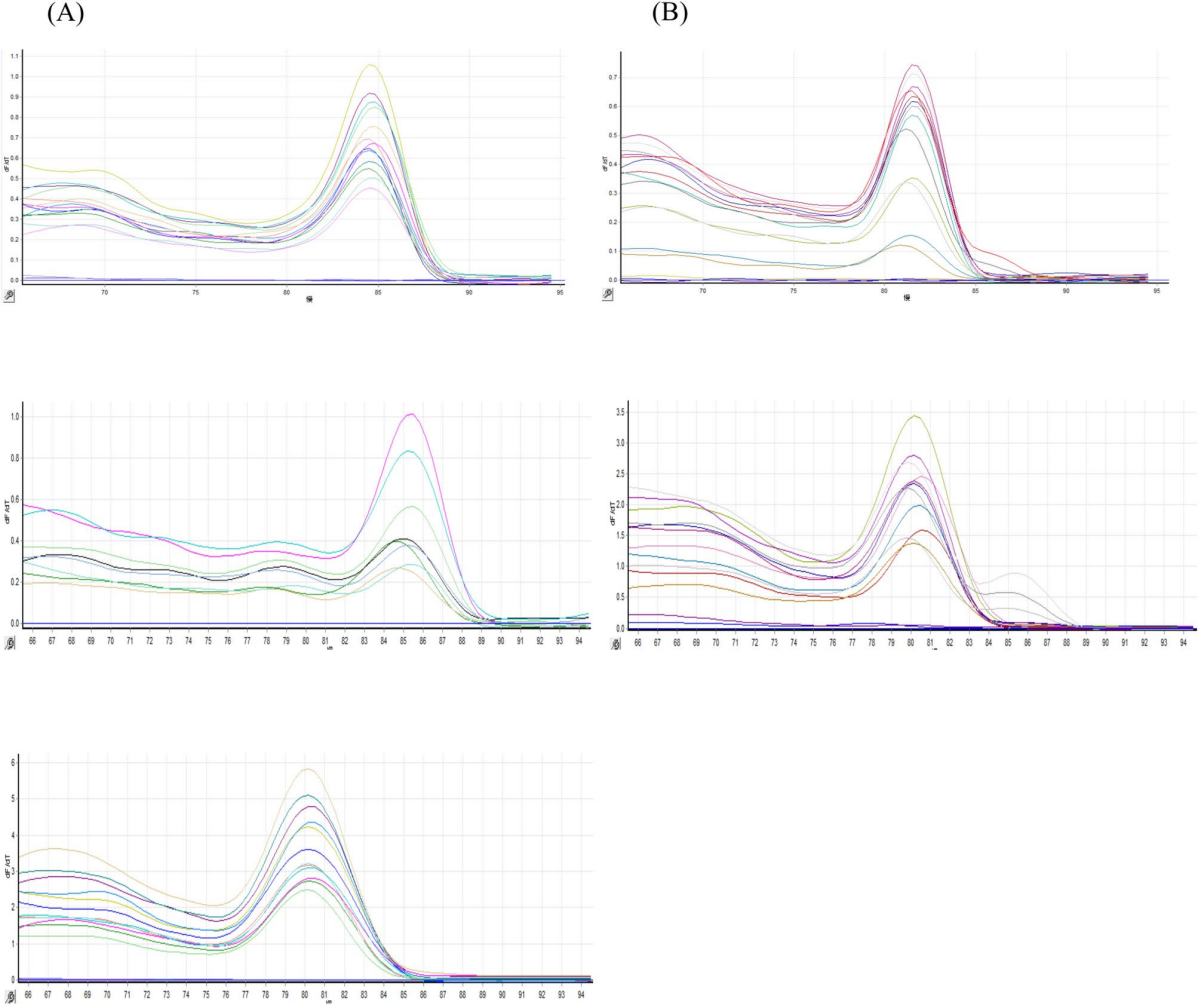
Melting curves of the examined genes, including (**A**) 18S rRNA, (**B**) ATPase, (**C**) β-actin, (**D**) gapdh, and (**E**) histone 3. The samples were chilled for various durations.

The efficiency of real-time PCR was validated by examining the melting curve (Fig. 3) generated for each amplification cycle of the five genes as follows: gapdh (Fig. 3D), 18S rRNA (Fig. 3A), ATPase (Fig. 3B), histone 3 (Fig. 3E), and β-actin (Fig. 3C). The efficiency of the primers and real-time PCR was confirmed by the single peak that appeared after each amplification cycle of the five tested genes. This finding indicates the absence of contamination and interference from primer dimers or misprinting.

Tables 2, 3 and 4 display the Ct values of all samples (Table 2) of the *S. hispidu*s embryos at various developmental stages (Table 3) and after various durations of chilling treatment (Table 4). The Ct value indicates the number of cycles required for a sample’s qPCR reaction to reach the critical threshold. The present study examined the Ct values of genes from multiple groups. Histone 3 exhibited the lowest Ct values (highest level of activity) among all samples (Table 2), indicating its role as an HKG. Conversely, 18S rRNA (23.25 ± 0.25) and β-actin (23.33 ± 0.74) had moderate Ct values. The Ct value of ATPase was the highest (31.89 ± 0.34), suggesting that the initial number of samples analyzed was the lowest, followed by gapdh (26.08 ± 0.88) (Table 2). The Ct value of histone 3 in the three developmental stages of the *S. hispidus* embryos was revealed to be the lowest (17.45 ± 0.34–20.39 ± 0.65), suggesting a high level of gene expression during the initial stage (Table 3). Conversely, the Ct value of ATPase was the highest (ranging from 30.27 ± 0.75 to 32.64 ± 0.51) across the three developmental stages of the *S. hispidus* embryos. The Ct values of the remaining three genes, namely 18S rRNA, β-actin, and gapdh, increased in the following order during the heartbeat period and pre-hatching stage: 18S rRNA (21.59 ± 0.20 to 22.54 ± 0.43), β-actin (23.08 ± 0.70 to 23.31 ± 1.41), and gapdh (25.96 ± 1.33 to 29.24 ± 1.35). However, when the eye was forming in the *S. hispidus* embryos, the Ct value of gapdh was lower than that of histone 3 (23.59 ± 1.71), and the Ct value of 18S rRNA (25.97 ± 0.33) was similarly lower than that of ATPase (32.64 ± 0.51) (Table 3). Furthermore, when the effects of chilling treatment on *S. hispidus* embryos at various time intervals were examined, the results indicated that the Ct values of histone 3 and ATPase were consistent with respect to their overall values (Table 2) and their values across various developmental stages (Table 3). Notably, histone 3 exhibited the lowest Ct value, ranging from 18.42 ± 1.06 to 19.13 ± 0.52 (Table 4). In the control group, the Ct values of β-actin were the second highest at 16 h (22.14 ± 1.41, 21.75 ± 1.79). For the genes that underwent chilling treatment for 4, 8, and 32 h, their Ct values ranged from 22.91 ± 2.14 to 25.89 ± 1.50, which placed them in the moderate range.

**Table 2:** Ct values for genes across all samples.

Gene	Ct
18S rRNA	23.25 ± 0.25
ATPase	31.89 ± 0.34
β-actin	23.33 ± 0.74
Gapdh	26.08 ± 0.88
Histone 3	18.82 ± 0.30

**Table 3:** Ct values of genes at various embryonic developmental stages of *S. hispidus*.

Gene	Eye-formation Ct	Heart-beat Ct	Pre-hatch Ct
18S rRNA	25.97 ± 0.33	21.59 ± 0.20	22.54 ± 0.43
ATPase	32.64 ± 0.51	32.45 ± 0.45	30.27 ± 0.75
β-actin	23.62 ± 1.66	23.08 ± 0.70	23.31 ± 1.41
Gapdh	23.59 ± 1.71	25.96 ± 1.33	29.24 ± 1.35
Histone 3	20.39 ± 0.65	17.45 ± 0.34	18.67 ± 0.39

**Table 4:** Effects of chilling on Ct values of genes after various durations of chilling.

Gene	Control	4 Hour	8 Hour	16 Hour	32 Hour
18S rRNA	23.48 ± 0.40	23.94 ± 0.43	23.59 ± 0.68	22.09 ± 0.65	22.67 ± 0.68
ATPase	31.91 ± 0.86	32.48 ± 0.77	32.12 ± 0.62	31.33 ± 0.71	31.22 ± 0.72
β-actin	22.14 ± 1.41	25.89 ± 1.50	23.34 ± 1.66	21.75 ± 1.79	22.91 ± 2.14
Gapdh	24.48 ± 1.79	27.58 ± 1.82	23.16 ± 2.04	29.21 ± 1.70	26.73 ± 2.43
Histone 3	18.67 ± 0.52	19.13 ± 0.52	19.04 ± 0.52	18.42 ± 1.06	18.69 ± 0.98

### Analysis of gene stability

**Fig. 4:**
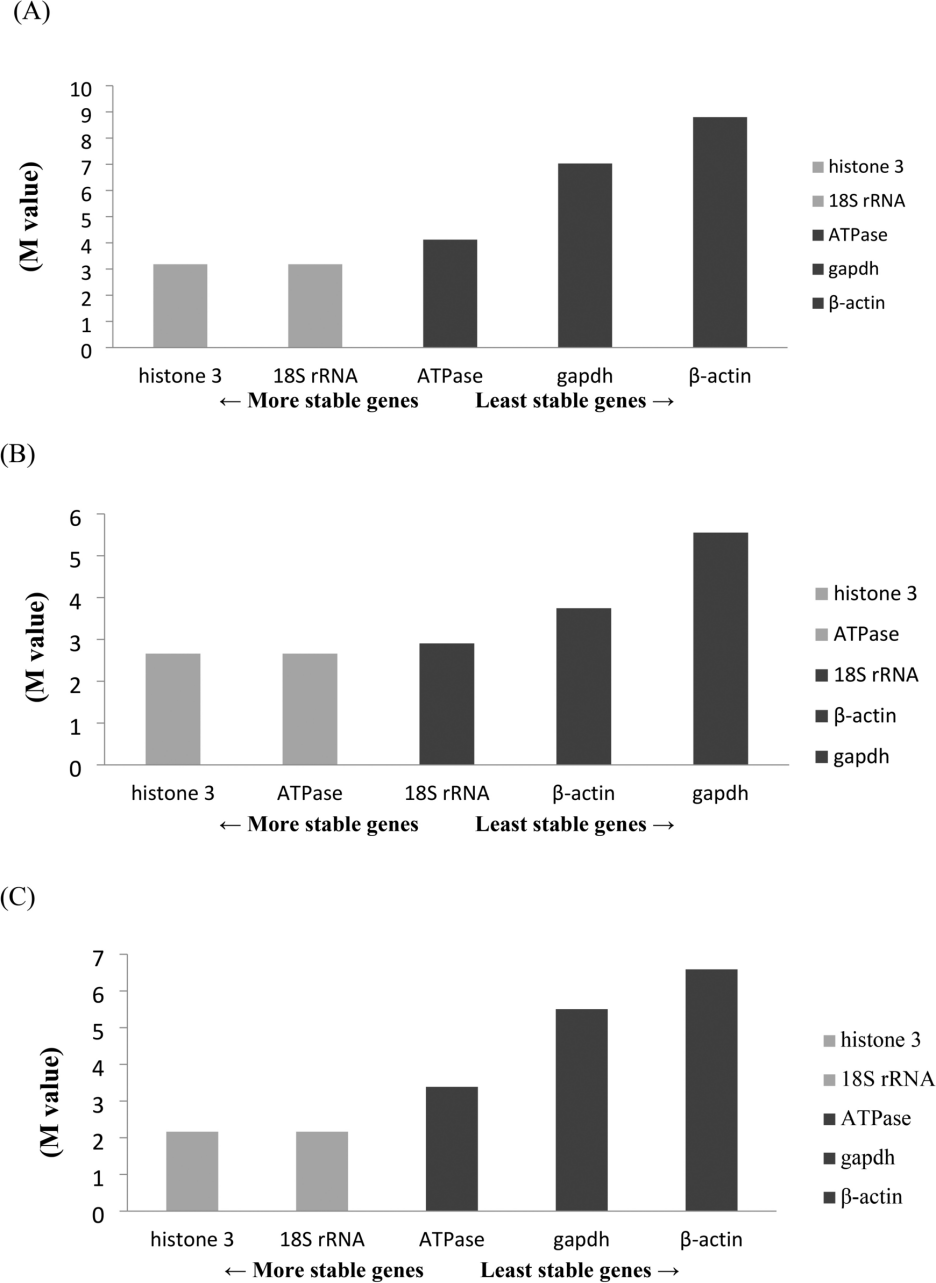
Use of the GeNorm algorithm to assess gene stability at various stages of embryonic development, namely the (**A**) eye formation, (**B**) heartbeat, and (**C**) pre-hatching stages. Genes exhibiting the highest stability are highlighted in gray.

**Fig. 5:**
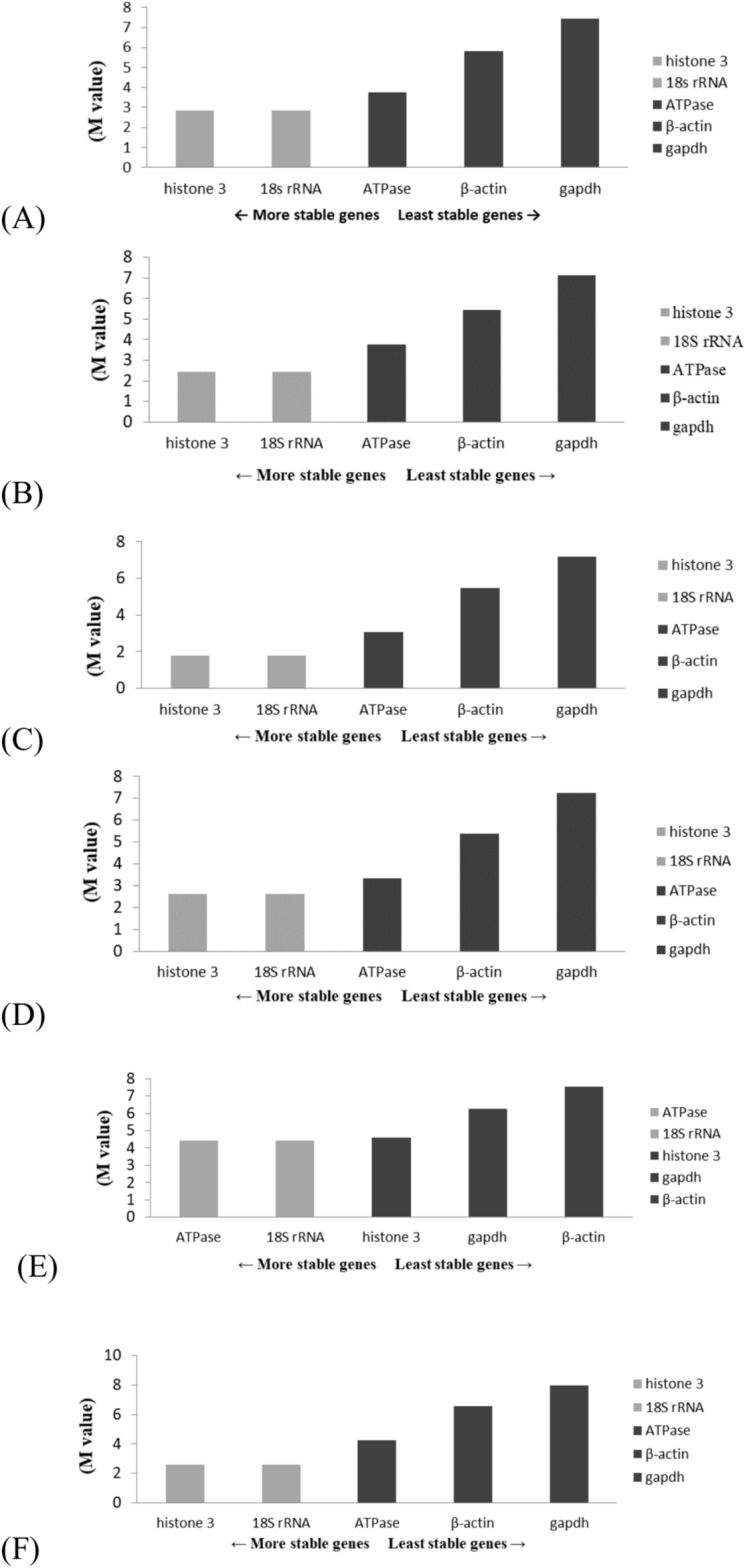
Use of the GeNorm algorithm to evaluate gene stability after various durations of chilling. (**A**) Entire set of samples; (**B**) chilled control group; (**C**) chilling for 4 h; (**D**) chilling for 8 h; (**E**) chilling for 16 h; (**F**) chilling for 32 h. Genes exhibiting the highest stability are highlighted in gray.

**Fig. 6:**
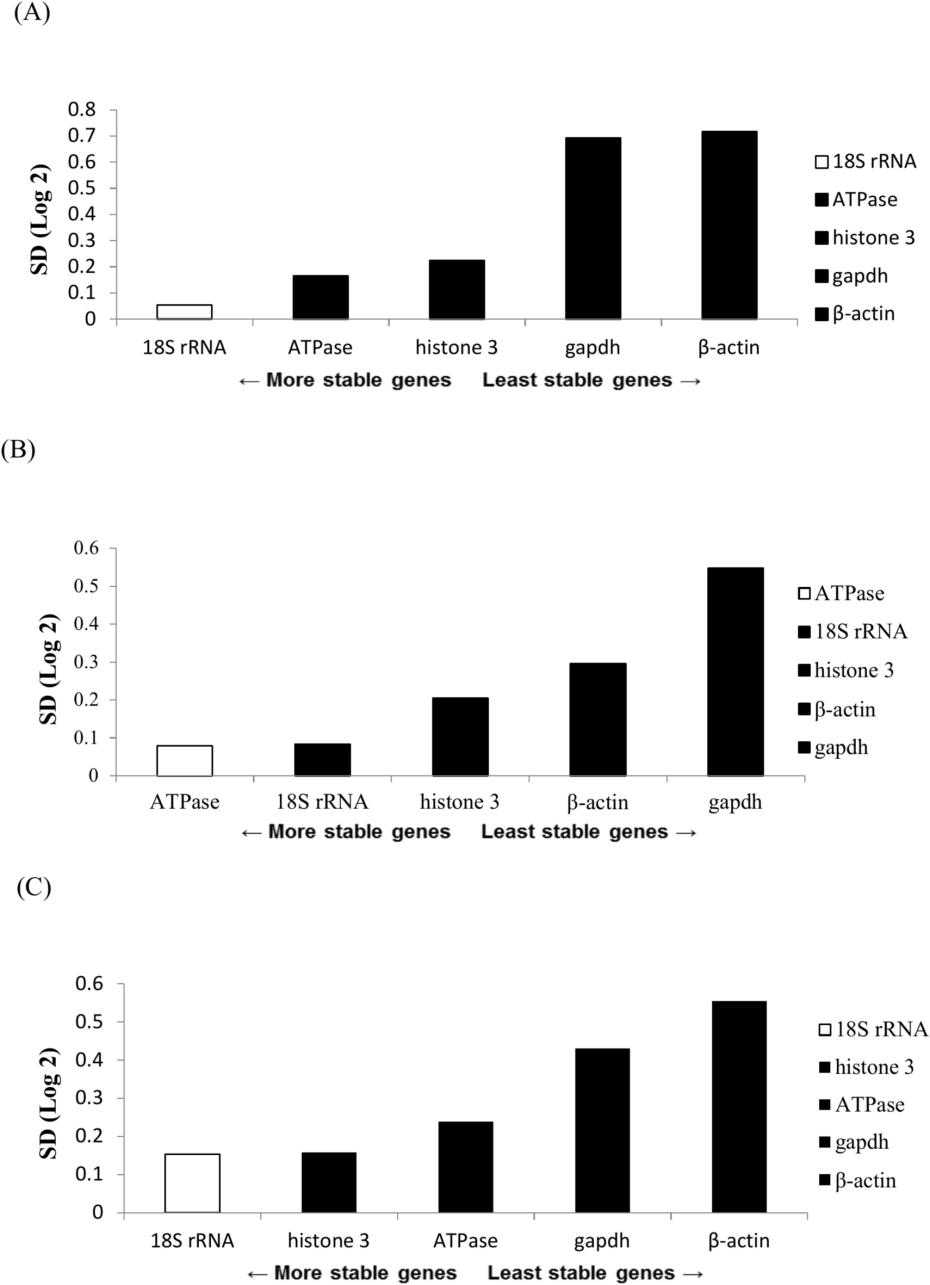
Use of the NormFinder algorithm to assess gene stability at various stages of embryonic development, namely the (**A**) eye formation, (**B**) heartbeat, and (**C**) pre-hatching stages. Genes exhibiting the highest stability are highlighted in white.

**Fig. 7:**
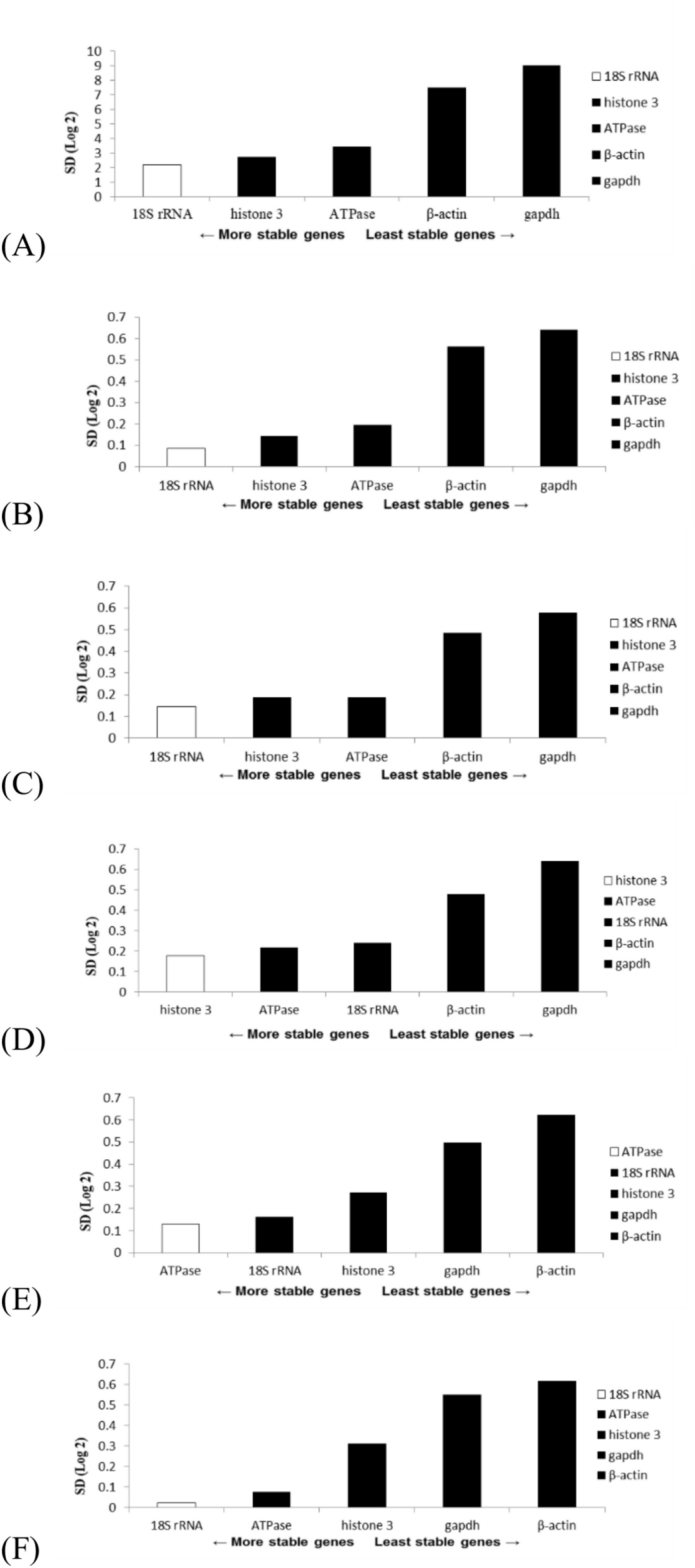
Use of the NormFinder algorithm to evaluate gene stability after various durations of chilling. (**A**) Entire set of samples; (**B**) chilled control group; (**C**) chilling for 4 h; (**D**) chilling for 8 h; (**E**) chilling for 16 h; (**F**) chilling for 32 h. Genes exhibiting the highest stability are highlighted in white.

The present study employed three algorithms, namely those in GeNorm, NormFinder, and Bestkeeper software, to assess the stability of the five genes. The GeNorm algorithm identified histone 3 and 18S rRNA as the most stable genes during the eye formation stage, heartbeat stage, and pre-hatching stage (Figs. 4A–C). Consistent findings were generally obtained for both the overall sample and the chilling groups at various time periods (Fig. 5A‒F); the only exceptions were the two most stable genes, ATPase and 18S rRNA (followed by histone 3, gapdh, and β-actin) after they were subjected to 16-h chilling treatment (Fig. 5E). The GeNorm algorithm identified GAPDH and β-actin as the most unstable genes. According to the NormFinder algorithm, the 18S rRNA gene was the most stable gene during the eye formation and pre-hatch stages (Figs. 6A,C). Among the genes studied during the heartbeat stage, ATPase exhibited the highest level of stability, followed by 18S rRNA, histone 3, β-actin, and gapdh, (Fig. 6B). Figure 7 presents the results obtained using the NormFinder algorithm to assess gene stability after various durations of chilling treatment. The 18S rRNA genes exhibited the highest level of stability in the control group and the groups that underwent 4 or 32 h of chilling treatment (Fig. 7A‒C and F). The outcomes for the samples after they were subjected to chilling treatment for 8 or 16 h are presented in Fig. 7D,E. These outcomes indicated that histone 3 and ATPase genes exhibited the highest level of stability. The NormFinder algorithm yielded results that were consistent with those obtained using the GeNorm algorithm, indicating that both gapdh and βactin exhibited the lowest stability across both control and chilling samples throughout the full duration of the experiment (Fig. 7A‒F).

In addition, the present study employed the Bestkeeper analysis software and compared its analysis outcomes with those obtained from the other two software programs. Notably, the results obtained through Bestkeeper diverged significantly from those obtained through GeNorm and NormFinder. The genes gapdh and β-actin were revealed to be the most stable in the overall sample and various embryonic stages and across the chilling periods (Tables 5, 6, 7). In contrast to prior findings, the GeNorm and NormFinder results indicated that histone 3 and 18S rRNA were the most stable; however, these results were not obtained when Bestkeeper was used (Tables 5, 6, 7).

**Table 5:** Evaluation of gene stability at various developmental stages of *S. hispidus* embryos through use of GeNorm, NormFinder, and Bestkeeper.

Stage	GeNorm		NormFinder		Bestkeeper	
Eye-formation	histone 3	1	18S rRNA	1	gapdh	1
	18S rRNA	1	ATPase	2	β-actin	2
	ATPase	3	histone 3	3	histone 3	3
	gapdh	4	gapdh	4	18S rRNA	4
	β-actin	5	β-actin	5	ATPase	5
Heart beat stage	histone 3	1	ATPase	1	gapdh	1
	ATPase	1	18S rRNA	2	β-actin	2
	18S rRNA	3	histone 3	3	ATPase	3
	β-actin	4	β-actin	4	18S rRNA	4
	gapdh	5	gapdh	5	histone 3	5
Pre-hatch stage	histone 3	1	18S rRNA	1	β-actin	1
	18S rRNA	1	histone 3	2	gapdh	2
	ATPase	3	ATPase	3	18S rRNA	3
	gapdh	4	gapdh	4	histone 3	4
	β-actin	5	β-actin	5	ATPase	5

**Table 6:** Comparison of results obtained using GeNorm, NormFinder, and Bestkeeper for analysis of gene stability after various durations of chilling.

Time	GeNorm		NormFinder		Bestkeeper	
Control	histone 3	1	18S rRNA	1	gapdh	1
	18S rRNA	1	histone 3	2	β-actin	2
	ATPase	3	ATPase	3	18S rRNA	3
	β-actin	4	β-actin	4	ATPase	4
	gapdh	5	gapdh	5	histone 3	5
4 Hours	histone 3	1	18S rRNA	1	gapdh	1
	18S rRNA	1	histone 3	2	β-actin	2
	ATPase	3	ATPase	3	ATPase	3
	β-actin	4	β-actin	4	histone 3	4
	gapdh	5	gapdh	5	18S rRNA	5
8 Hours	histone 3	1	histone 3	1	β-actin	1
	18S rRNA	1	ATPase	2	gapdh	2
	ATPase	3	18S rRNA	3	histone 3	3
	β-actin	4	β-actin	4	18S rRNA	4
	gapdh	5	gapdh	5	ATPase	5
16 Hours	histone 3	1	18S rRNA	1	β-actin	1
	18S rRNA	1	histone 3	2	gapdh	2
	ATPase	3	ATPase	3	18S rRNA	3
	gapdh	4	gapdh	4	histone 3	4
	β-actin	5	β-actin	5	ATPase	5
32 Hours	histone 3	1	18S rRNA	1	gapdh	1
	18S rRNA	1	ATPase	2	β-actin	2
	ATPase	3	histone 3	3	18S rRNA	3
	β-actin	4	gapdh	4	histone 3	4
	gapdh	5	β-actin	5	ATPase	5

**Table 7:** Evaluation of genetic stability of overall sample through use of GeNorm, NormFinder, and Bestkeeper.

	GeNorm		NormFinder		Bestkeeper	
All samples	Histone 3	1	18S rRNA	1	Gapdh	1
	18S rRNA	1	Histone 3	2	β-actin	2
	ATPase	3	ATPase	3	Histone 3	3
	β-actin	4	β-actin	4	18S rRNA	4
	Gapdh	5	Gapdh	5	ATPase	5

## Discussion

This study examined five HKGs, namely gapdh, 18S rRNA, histone 3, ATPase, and β-actin. In organisms, gene expression signifies the regulation of cellular processes, and its quantification is frequently employed as a biological and molecular indicator. Researchers have increasingly focused on the effects of gene expression in a wide variety of species, including mammals^71,72^, algae^73,74^, mollusks^5,75^, and fish^3,76,77^.

Crustacean research has focused mainly on investigating virus immunological mechanisms and reproductive systems^8,78–81^. At present, the research on cryopreservation in shrimp species is limited, and no study has explored the effect of cryopreservation on gene expression. The first research report on *S. hispidus* was published by Olivier in 1811. Subsequent studies have primarily focused on topics such as growth and development, characteristic behaviors, breeding enhancement, genetic attribute classification, and the geographical distribution of *S. hispidus*^67,82–88^. The enzymatic separation method was proposed by Tsai and Lin 2009^89^, and this method has facilitated the effective extraction of individual embryos for subsequent investigations. Subsequently, efforts have been made to develop cryopreservation techniques, which involve assessing the effects of cryoprotectants and exposure to low temperatures^70,73^. Accordingly, the present study determined whether low-temperature chilling affects gene expression, which is a crucial factor in regulating cellular processes in living organisms. To this end, the first step involved investigating the most appropriate HKGs for gene expression analysis.

The present study determined the presence of five genes, namely 18S rRNA, histone 3, ATPase, β-actin, and gapdh. The first essential step was to verify primer specificity for *S. hispidus* genes. Specific primers were constructed for *S. hispidus* on the basis of the known sequences of 18S rRNA, histone 3, and ATPase. For β-actin and gapdh, we designed their primers by referencing other shrimp species because no published sequences are currently available. On the basis of our research, conventional PCR and gel electrophoresis were able to accurately amplify the targeted sequences by using the designed primers. In 2015, García et al.^90^ employed this method to investigate viruses that cause infectious subcutaneous and hematopoietic tissue necrosis in *Penaeus stylirostris*. Additionally, Chiou et al. 2007^91^ and Ho and Song 2009^92^ employed this method to identify and analyze various shrimp species. In addition, the determination of the melting temperature of double-stranded DNA products was performed using the amplification melting curve obtained from quantitative real-time PCR. In contrast to other studies that have used specific probes, our research employed SYBR green to excite the fluorescence in DNA double-stranded helices^93,94^. This method resulted in a single peak curve, indicating the absence of contamination caused by primer dimmers or errors in real-time PCR reactions^8,22,95^.

Techniques for detecting gene expression include microarray, SAGE, ISH, Northern blotting, and others. The choice of technique is mostly determined by the conditions and detection requirements of a given set of samples. Microarray and SAGE are effective methods for simultaneously detecting a substantial number of samples from various sources or treatments, which is required to analyze gene functions^65^. SAGE analysis is primarily used to identify novel genes^96^. The ISH technique may be employed to identify individual sequences of specific nucleic acids and to determine locations of cellular expression^97^. Northern blotting is frequently employed to ascertain the presence of genetic diversity in a sample gene^98^. However, a drawback of this method is the laborious process of RNA preparation. The technique employed in the present study, quantitative real-time PCR, differs from conventional PCR in that it does not require electrophoresis analysis. Quantitative real-time PCR involves the application of fluorescence technology to detect fluorescent signals emitted by DNA, allowing for the number of cycles (Ct value) to be determined. Quantitative real-time PCR has been employed to investigate gene expression in various species^4,11^, such as zebrafish blastermeres and tissues^1,99^, shrimp germ cells and gametes^8,34,100,101^, tilapia genetic correlation^102^, algal endosymbiotic systems^9,103,104^, and human respiratory coronaviruses^105–107^.

In quantitative real-time PCR, the Ct value is commonly employed to assess the relative expression of housekeeping genes and target genes (ΔCt)^108,109^. In general, the Ct value allows for an approximate estimation of the gene transcription for each gene^1^. No specific ideal range for Ct values has been established. Typically, only genetic variations within the same set of samples are evaluated. The association between Ct levels and gene expression is inversely linked^110,111^. The mean Ct values obtained in the present study, presented in ascending order, were 17–20 for histone 3, 21–23 for 18S rRNA, and 30–32 for ATPase. Notably, the present study is the first to investigate and reveal the expression of histone 3 in shrimp. The gene expression of ATPase was investigated in another study that examined the effects of various environmental stresses, such as low temperature stress, on white shrimp^101^. Its findings revealed a decrease in ATPase expression when white shrimp were subjected to these stresses, which aligns with the results of our study.

The suitability and stability of HKGs are frequently discussed in the context of gene expression^2,3,8,10,13,54^. Using the GeNorm algorithm, we identified histone 3 and 18S rRNA as the most stable HKGs. According to the NormFinder results, 18S rRNA was the most stable HKG. Histone 3 is an essential nuclear protein found in eukaryotes^112^. It plays a crucial role in forming the nucleosome structure of the chromosomal fiber. Studies conducted in 2009 and 2011 have demonstrated that histone 3 plays a primary role in changing chromatin to establish the structure of chromosomes throughout the process of eukaryotic cell division. Additionally, histone 3 is a protein that regulates the process of DNA folding. It exhibits resistance to cell mutations^113,114^ and undergoes phosphorylation alteration throughout the early embryonic stages of mouse sperm cells^115^. Histone 3 has already been employed to determine the evolutionary relationships of crustacean taxonomy^116^, including the genus Stenopus spp.^117^, which was the experimental species in the present study. By contrast, 18S rRNA is a component of the ribosomal RNA unit, and it primarily facilitates the process of intracellular translation^118^. Additionally, 18S rRNA has been used as an HKG in gene expression research. These genes have also been used as HKGs in animals and plants^5,11,119–123^. The literature on Japanese shrimp HKGs indicates that 18S rRNA remains stable throughout shrimp development and that it can serve as a reliable HKG during the developmental cycle of shrimp^12^. Ruan and Lai 2007^124^ made a similar assertion. By contrast, Leelatanawit et al. 2012^8^ studied HKGs associated with the reproductive system of grass shrimp, and they suggested that 18S rRNA is lost during mRNA extraction, thereby affecting the outcomes of quantitative real-time PCR analysis. Furthermore, if the level of gene expression of a gene is high, such as in the case of 18S rRNA, the gene should not be used as an HKG.

gapdh and β-actin are commonly employed as internal HKGs or protein analysis standards in numerous gene expression-related research studies, including those involving shrimp, mammals, and fish^100,125–129^. Nevertheless, in our study, GeNorm and NormFinder identified gapdh as the most unstable HKG, with β-actin being the second most unstable one. These findings are consistent with those of several studies. The primary role of GAPDH is to control intracellular glycolysis^130,131^. Leelatanawit et al. 2012^8^ and Ruan et al. 2007^124^ discovered that gene expression undergoes alterations during the developmental phase of individual cells and in response to hormonal stimulation. Conversely, β-actin is mainly responsible for encoding cytoskeletal structural proteins^12,124,132^ demonstrated that the gene expression levels in Penaeus japonicus embryos vary across developmental stages. Selvey et al. 2001^133^ argued that β-actin is unsuitable for use as an HKG. They contended that the cell culture matrix influences the expression of β-actin and advocated the use of 18S rRNA as a more suitable gene for normalizing gene expression^133^. Additionally, an investigation of mouse serum fibroblast revealed that the regulation of gapdh and β-actin changed depending on the duration of sample operation, making them unsuitable as HKGs^11^. On a positive note, gapdh is an ideal HKG for researching immunological infection in tiger shrimp (i.e., *Penaeus monodon*^4^). Notably, in the present study, Bestkeeper revealed that gapdh was the most stable among the five evaluated HKGs. However, in a 2008 study that employed Bestkeeper, the analysis was limited by experimental conditions and required a standard deviation of less than 1, which is only suitable for small sample sizes. During an evaluation of a considerable quantity of samples, mistakes are likely to occur^59^. The sample size in the present study was greater than 10, suggesting that the use of gapdh as an HKG should be avoided in our future studies.

In organisms, ion transport across cell membranes is primarily facilitated by Na+/K+-ATPase (NaK), which plays a crucial role in controlling cell volume, cell membrane potential, and several other biological mechanisms^134–136^. In their 2012 study, Wang et al. 2012^135^ were the first to elucidate the role of ATPase in shrimps and its effect on gene expression under varying levels of pressure. They discovered that exposure to high salinity led to more pronounced variations in ATPase expression levels in the gill and liver tissues relative to other tissues. The ATPase enzyme is strongly associated with shrimp osmotic pressure, energy storage, and toxin metabolism, as demonstrated by Wang et al. 2012^135^, who provided insights into the distinct behavior of ATPase in response to environmental stress. Our research findings indicate that ATPase is unsuitable for use as an HKG in a low temperature study. However, it can serve as a target gene for investigating cell damage caused by environmental stress in future studies.

Our study involved exposing *S. hispidus* embryos at various developmental stages to prolonged durations of low-temperature chilling. Our analysis of optimal gene stability revealed no notable disparity in the experimental samples relative to the full set of samples. In their 2004 study, Kim and Lee^137^ suggested that environmental stress causes DNA damage during embryonic development, with the effect being greater in early-stage embryos than in later-stage embryos. Alfaro et al. 2001^138^ demonstrated that larvae are more resistant to low temperatures and cryoprotectants relative to embryos. The 1998 and 2013 studies (Gwo and Lin 1998^139^; Lin et al. 2013^88^) also reached the same conclusion. An egg yolk primarily serves as a source of energy for the growth and development of an embryo. Freshwater shrimp embryos, similar to their ocean counterparts, require yolks as a source of nutrition^140^. A yolk’s content and composition can influence the low-temperature tolerance of shrimp. By contrast, various studies have examined the immune-related systems of shrimp and determined that late-stage embryos and larvae have completely developed immune systems, resulting in enhanced resistance to microbial infections^141^.

Our study identified histone3 and 18S rRNA as suitable HKGs for studying the effects of low temperature on *S. hispidus* embryos. These genes were ranked as the most suitable HKGs by GeNorm and NormFinder. Although ATPase may not be a reliable HKG, we propose that it can serve as a suitable target gene for studying the metabolism or physiological condition of shrimp species. Similarly, both gapdh and β-actin are not stable HKGs for low-temperature studies. Notably, gapdh can be used as a basis for HKGs in infection-related research, whereas β-actin can be used as a target gene for the production of cell growth genes. Nevertheless, these hypotheses must be verified through additional investigation. Our study is the first to investigate the suitable HKGs for studying the effect of low-temperature treatment on shrimp species. Although additional suitable genes may exist, the present study has made a pioneering contribution toward future research on the effect of cryopreservation on gene expression in shrimp embryos.

## Supplementary Information

Supplementary Information 1.

Supplementary Information 2.

Supplementary Information 3.

Supplementary Information 4.

Supplementary Information 5.

Supplementary Information 6.

Supplementary Information 7.

Supplementary Information 8.

Supplementary Information 9.

Supplementary Information 10.

Supplementary Information 11.

Supplementary Information 12.

## Data Availability

Data is provided within supplementary information files.
